# Low-Rank and Sparse Decomposition Model for Accelerating Dynamic MRI Reconstruction

**DOI:** 10.1155/2017/9856058

**Published:** 2017-08-08

**Authors:** Junbo Chen, Shouyin Liu, Min Huang

**Affiliations:** ^1^College of Physical Science and Technology, Central China Normal University, Wuhan 430079, Hubei, China; ^2^Key Laboratory of Cognitive Science of State Ethnic Affairs Commission, South-Central University for Nationalities, Wuhan 430074, Hubei, China; ^3^Hubei Key Laboatory of Medical Information Analysis & Tumor Diagnosis and Treatment, Wuhan 430074, Hubei, China

## Abstract

The reconstruction of dynamic magnetic resonance imaging (dMRI) from partially sampled *k*-space data has to deal with a trade-off between the spatial resolution and temporal resolution. In this paper, a low-rank and sparse decomposition model is introduced to resolve this issue, which is formulated as an inverse problem regularized by robust principal component analysis (RPCA). The inverse problem can be solved by convex optimization method. We propose a scalable and fast algorithm based on the inexact augmented Lagrange multipliers (IALM) to carry out the convex optimization. The experimental results demonstrate that our proposed algorithm can achieve superior reconstruction quality and faster reconstruction speed in cardiac cine image compared to existing state-of-art reconstruction methods.

## 1. Introduction

Dynamic MRI (magnetic resonance imaging), an essential medical imaging technique, allows noninvasiveness, nonionization visualization, and analysis of anatomical and functional changes of internal body structure through time. However, MRI sampling speed is relatively slow due to the need of physical and physiological conditions such as nuclear relaxation and peripheral nerve stimulation [1]. One way for accelerating MRI is to reconstruct high-resolution images from undersampled *k*-space data. However, such undersampling violates the Nyquist criterion and often results in aliasing artifacts if the traditional linear reconstruction is directly applied.

To address this issue, there are so much research efforts to accelerate MRI acquisition process using hardware and software [2–4]. Among them, compressed sensing (CS) has been proved to be able to increase imaging speed and efficiency in MRI application [5–7]. The CS theory requires image sparsity and incoherence between the acquisition space and representation space [8]. Fortunately, the MR image sequence often provides redundant information in both spatial and temporal domains, which presents favorable conditions for the application of CS. In addition, the idea is easily extended to the reconstruction of dynamic MRI (dMRI) images due to extensive spatio-temporal correlations that result in sparser representations. The *k-t* FOCUSS is a successful method, which imposes a sparsity constraint in the temporal transform domain by using the FOCUSS algorithm [9], and extends the FOCUSS technique with motion estimation and compensation to compressed sensing framework for cardiac cine MRI. But the limitation of the prediction schemes on sparsifying the residual signal sets back the further improvement when the motion is aperiodic.

Recently, researchers have made great efforts to exploit the low-rank property of matrices instead of simply sparsity of vectors. Lingala et al. proposed a *k-t* SLR algorithm that exploited the low rank prior and global sparsity in Karhunen-Louve Transform (KLT) domain for MRI reconstruction [10]. However, the algorithm failed to take into account the structural sparsity of the MRI image, and the limitation held back the further improvement. Some studies presented patch-based dictionary learning techniques for dMRI reconstruction [11, 12]. However, a major challenge in learning sparse dictionary is that such patch-based learning cannot be effectively employed for dMRI reconstruction. Because the size of dMRI sequence is large, it is inefficient to learn dictionaries for such large datasets [13]. Even though we take no account of computational limitations, it is not practical to acquire such huge dMRI training sequences for learning sparsifying dictionaries. Currently, robust principal component analysis (RPCA) has been used in recovering dynamic images to explore the low-rank structure of data [14, 15]. The RPCA decomposes the data in low rank and sparse components, where the low rank component models the temporally correlated background information and the sparse component represents the dynamic information. *k-t* RPCA [16], a method developed for dMRI, uses the low-rank plus sparse decomposition prior to reconstructing dynamic MRI from part of the *k*-space measurements. In this method [16], the image reconstruction is regularized by a low-rank plus sparse prior, where the Fourier transform is used as the sparsifying transform and the alternating direction methods of multipliers (ADMM) is applied to solve the minimization problem in the temporal direction. The shortcoming of *k-t* RPCA is that the results of reconstructed image are easily affected by the noise, since the noise will generally be represented by highly sparse coefficients during the sparsifying transform.

In this paper, aims to the shortcoming of *k-t* RPCA, we propose an efficient numerical algorithm based on inexact augmented Lagrangian method (IALM) instead of ADMM to solve the optimization problem and accelerate the dMRI reconstruction. The experimental results demonstrate that our proposed algorithm can achieve more satisfactory reconstruction performance and faster reconstruction speed in given cardiac cine sets.

## 2. Theory Background

The dynamic MRI data acquisition in the *k-t* space can be expressed as follows:

(1) $$\begin{array}{c} y\left(k,t\right)=\int x\left(r,t\right)\exp\left(-2\pi jk\;\cdot \;r\right)dr+n\left(k,t\right), \end{array}$$

where *y*(*k*, *t*) represents the measured *k-t* space signal, *x*(*r*, *t*) denotes the desired dynamic image series, and *n*(*k*, *t*) is the measured noise, which can be reasonably modeled by an additive white Gaussian distribution [16, 17].

In this paper, the solution of this problem is to find the closest representation of the MR image *x*(*r*, *t*) from undersampled measurement *y*(*k*, *t*). Since the *k-t* space is partially sampled, (1) is converted to an inverse problem and can be rewritten as a vector [18].

(2) $$\begin{array}{c} Y=RFX+n, \end{array}$$

where **Y** = [*y*_1_ | ⋯∣*y*_*T*_], **X** = [*x*_1_ | ⋯∣*x*_*T*_], **n** = [*n*_1_ | ⋯∣*n*_*T*_], *T* is the total number of frames, *F* is the Fourier transform operator, and the measurement matrix **R** is the undersampled mask applied on the *k*-space.

### 2.1. CS-Based MR Image Reconstruction

The CS approach [5, 19] was proposed to reconstruct the MR image **X** from the partially sampled *k*-space data **Y** by exploiting the sparsity transform and convex optimization algorithms. The problem will be solved if we can find the sparsest vector satisfying (2),

(3) $$\begin{array}{c} \begin{array}{ccc} \underset{X}{\min}{\left\|DX\right\|}_{0} & s.t. & {\left\|Y-RFX\right\|}_{2}\leq \varepsilon , \end{array} \end{array}$$

where ‖·‖_0_ is *l*_0_-norm, counting the number of nonzero entries in the vector, *D* is the sparsifying transform or dictionary, and *ε* is a small constant. Unfortunately, (3) is NP-hard problem, which needs to be solved by a brute force search. The CS theory [8] proves that the convex relaxation approach referred to as *l_1_* minimization can be replaced with the *l*_0_-norm in (3),

(4) $$\begin{array}{c} \begin{array}{ccc} \underset{X}{\min}{\left\|DX\right\|}_{1} & s.t. & {\left\|Y-RFX\right\|}_{2}\leq \varepsilon , \end{array} \end{array}$$

where ‖·‖_1_ is *l*_1_-norm, meaning the sum of absolute values of the vector.

### 2.2. Low-Rank and Sparse Decomposition Model for MR Image Reconstruction

CS-based techniques that exploit sparsity of the image in the transform domain have been successfully used for MR image reconstruction. However, the performance of CS is primarily dependent on the specific dictionary or sparsifying operator, which limits the maximum achievable acceleration rate. Therefore, some researchers tried to investigate a few new approaches to reconstruct MR image [20–24]. In those methods, low-rank matrix recovery is a popular technique in medical image processing.

The basic assumption is the same as [18], that is, the image **X** is simultaneously sparse (in a transform domain) and low rank. The problem is to recover **X**, given fewer *k*-space samples **Y** than the number of elements in the matrix. We assume that the approximate rank of the matrix is *r* and the size of single frame image is *M* × *N*. When the matrix **X** is low rank, which has only *r*(*M* + *N* − *r*) degrees of freedom instead of *MN*, it is possible to recover the matrix **X** from lesser number of samples by solving the rank minimization problem,

(5) $$\begin{array}{c} \begin{array}{ccc} \underset{X}{\min}\;\mathrm{rank}\left(X\right) & s.t. & {\left\|Y-M\left(X\right)\right\|}_{2}\leq \varepsilon . \end{array} \end{array}$$

However, the rank minimization problem, that is, solving (5), is combinatorial and known to be NP-hard [25]. Therefore, convex relaxation is often used to make the minimization tractable.

(6) $$\begin{array}{c} \begin{array}{ccc} \underset{X}{\min}{\left\|X\right\|}_{*} & s.t. & {\left\|Y-M\left(X\right)\right\|}_{2}\leq \varepsilon , \end{array} \end{array}$$

where *M* denotes any linear operator and ‖**X**‖_∗_ is the nuclear norm, which is defined as

(7) $$\begin{array}{c} {\left\|X\right\|}_{*}=\overset{r}{\underset{i=1}{\sum }}\sigma_{i}, \end{array}$$

where *σ*_1_, *σ*_2_,…, *σ*_*r*_ are the singular values of **X** and *r* is the rank of **X**.

To recover **X** from the given **Y**, **X** can be decomposed into a superposition of a low-rank matrix **A** and a sparse matrix **E**.

(8) $$\begin{array}{c} X=A+E. \end{array}$$

**X** is recovered as the solution of the following optimization:

(9) $$\begin{array}{c} \begin{array}{ccc} \underset{A,E}{\min}{\left\|A\right\|}_{*}+\gamma {\left\|E\right\|}_{1} & s.t. & {\left\|Y-M\left(A+E\right)\right\|}_{2}\leq \varepsilon \end{array}, \end{array}$$

where low-rank matrix **A** has few nonzero singular values and represents the background component, sparse matrix **E** has few nonzero entries and corresponds to the changes, and *γ* is a tuning parameter that balances the contribution of the *l*_1_-norm relative to the nuclear norm.

## 3. The Proposed Method

In principal component pursuit (PCP) model [26], to solve (9) can be posed as an optimization problem by using regularization rather than strict constraints [15]. Hence, (9) can be converted as

(10) $$\begin{array}{c} \underset{L,S}{\min}{{\left\|Y-M\left(A+E\right)\right\|}_{F}^{2}+\lambda_{L}\left\|A\right\|}_{*}+\lambda_{S}{\left\|TE\right\|}_{1}, \end{array}$$

where the parameters *λ*_*L*_ and *λ*_*S*_ trade off data consistency and *T* is a sparse transform basis.

Equation (10) is a RPCA problem that involves minimizing a combination of the nuclear norm and *l_1_*-norm. Otazo et al. Study [15] adopted the iterative thresholding scheme to solve (10); however, the iterative thresholding technique converges slowly. So, we presented an inexact augmented Lagrange multipliers (IALM) algorithm to solve the RPCA problem [27]. According to the constraint conditions of (6),

(11) $$\begin{array}{c} M^{H}y=A^{\left(n\right)}+E^{\left(n\right)}=X^{\left(n\right)}, \end{array}$$

where *M*^*H*^ is a dual operator, **X**^(*n*)^ contains the measurement noise, and **A**^(*n*)^ and **E**^(*n*)^ are low-rank element and sparse element, respectively. We applied IALM method to solve the following optimization problem:

(12) $$\begin{array}{c} \underset{L,S}{\min}{\left\|A^{\left(n\right)}\right\|}_{*}+\lambda {\left\|TE^{\left(n\right)}\right\|}_{1}+\langle L,M^{H}y-A^{\left(n\right)}-E^{\left(n\right)}\;\rangle +\frac{\mu }{2}{\left\|M^{H}y-A^{\left(n\right)}-E^{\left(n\right)}\right\|}_{F}^{2}, \end{array}$$

where *ℒ* is a Lagrange multiplier to remove the equality constraint and *μ* is a small positive scalar. The condition $\overset{+\infty }{\underset{k=1}{\sum }}\mu_{k}^{-1}=+\infty$ implies that *μ*_*k*_ cannot grow too fast. The IALM method for solving the RPCA problem can be described as Algorithm 1.

For Algorithm 1, if {*μ*_*k*_} is nondecreasing and $\overset{+\infty }{\underset{k=1}{\sum }}\mu_{k}^{-1}=+\infty$, then (**A**_*k*_, **E**_*k*_) converges to an optimal solution (**A**^∗^, **E**^∗^) for the RPCA problem. The advantage of unbounded {*μ*_*k*_} is that the feasibility condition **A**_*k*_ + **E**_*k*_ = **X** can be approached more quickly because **X** − **A**_*k*_ − **E**_*k*_ = (*ℒ*_*k*_ − *ℒ*_*k*−1_)/*μ*_*k*−1_ and {*ℒ*_*k*_} are bounded. In Algorithm 1, the singular value thresholding (SVT) operator [28] is defined as

(13) $$\begin{array}{c} SVT_{\lambda }\left(D\right)=U\Lambda_{\lambda }\left(\Sigma \right)V^{H}, \end{array}$$

where **D** = *U*Σ*V*^*H*^ is any singular value decomposition of **D**. *Λ*_*λ*_(Σ) is a soft-thresholding operator, which can be defined as

(14) $$\begin{array}{c} \Lambda_{\lambda }\left(x\right)=\frac{x}{\left|x\right|}\max\left(\left|x\right|-\lambda ,0\right). \end{array}$$

## 4. Experimental Results and Discussion

**Figure 1:**
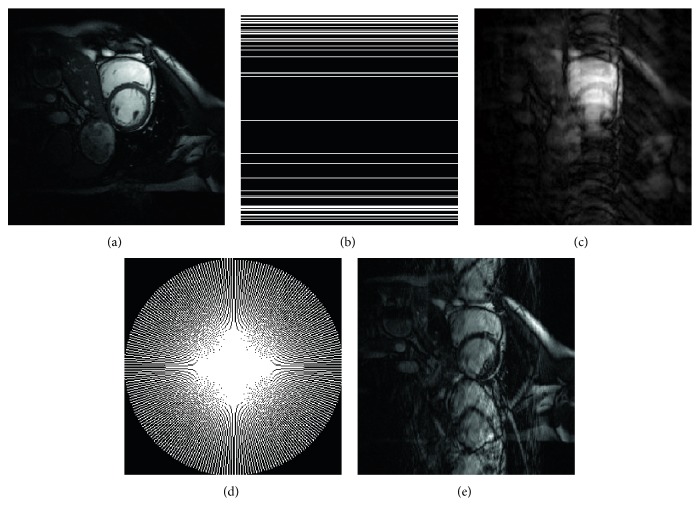
Example of two undersampling masks and their effect on reconstruction image. (a) A magnitude temporal frame from one of the cardiac cine datasets. (b) The Cartesian undersampling mask for a magnitude temporal frame. (c) The zero-filled inverse Fourier transform reconstruction image. (d) The pseudo-radial sampling acquisition for a magnitude temporal frame. (e) The zero-filled inverse Fourier transform reconstruction image.

Experiments were run in MATLAB V7.14.0 (R2012a) with the computing environment being an Intel Core i7-2640 M CPU, 4.0 GB memory, and a 64-bit Win7 operating system. The proposed algorithm was validated by experiments using two cardiac cine sets. The first dataset was obtained from Bio Imaging and Signal Processing Lab (http://bispl.weebly.com/), which contains *n*_*t*_ = 25 temporal frames of size *n*_*x*_ = *n*_*y*_ = 256 with a 345 × 270 mm^2^ field of view (FOV) and 10 mm slice thickness. The second dataset was acquired from the website of Dr. Caballero (http://www.doc.ic.ac.uk/~jc1006/index.html), which was introduced by Caballero et al. [12] and the relevant imaging parameters were as follows: the image matrix size = 256 × 256  (*n*_*x*_ × *n*_*y*_), the number of temporal frame = 30 (*n*_*t*_), FOV = 320 × 320 mm^2^, and slice thickness =10 mm. Two widely used sampling trajectories, Cartesian and radial undersampling strategies, were exploited for the acquisition of the MR data set in the *k*-space domain.  Figure 1 shows the sampling masks used in the study and their effect on the magnitude of a temporal frame.

We compared the proposed method against *k-t* SLR [10] and *k-t* RPCA [16] in reconstruction accuracy and reconstruction speed. Quantitative image quality assessment was performed by using the metrics of peak signal to noise ratio (PSNR) and structural similarity index (SSIM) [29]. The PSNR is used to evaluate the difference of reconstruction image and the full-sampled image, which can be defined as

(15) $$\begin{array}{c} PSNR=-10\log_{10}\left(\frac{{\left\|\hat{X}-X\right\|}_{F}^{2}}{{\left\|X\right\|}_{F}^{2}}\right), \end{array}$$

where $\hat{X}$ is a reconstruction image and **X** represents the full-sampled image.

The SSIM is a new method for measuring the similarity between the reconstructed image and the fully sampled image. We adopted the SSIM to measure the difference of reconstruction image and the fully sampled image at each time frame {*x*_*n*_}_*n*=1_^*n*_*t*_^; the SSIM index between the reconstructed image *x*_Rec_ and the fully sampled image *x*_*F*_ at one same frame is evaluated as

(16) $$\begin{array}{c} SSIM\left(x,y\right)=\frac{\left(2\mu_{x_{R}}\mu_{x_{F}}+c_{1}\right)\left(2\sigma_{x_{R}x_{F}}+c_{2}\right)}{\left(\mu_{x_{R}}^{2}+\mu_{x_{F}}^{2}+c_{1}\right)\left(\sigma_{x_{R}}^{2}+\sigma_{x_{F}}^{2}+c_{2}\right)}, \end{array}$$

where *μ*_*x*_*R*__ and *μ*_*x*_*F*__ are the mean intensity of the reconstructed image *x*_Rec_ and the fully sampled image *x*_*F*__,_*σ*_*x*_*R*__ and *σ*_*x*_*F*__ are the standard deviation of image *x*_Rec_ and *x*_*F*_, *σ*_*x*_*R*_*x*_*F*__ is the covariance of *x*_Rec_ and *x*_*F*_, *c*_1_ = (*K*_1_*L*)^2^ and *c*_2_ = (*K*_2_*L*)^2^ are constants where *L* is the dynamic range, 255 for 8-bit grayscale images. *K*_1_ = 0.01 and *K*_2_ = 0.03 are parameter values suggested by Wang et al. [29].

The *k-t* SLR uses a combination of TV and nonconvex Schatten p-norms with *p* = 0.01; some parameters are selected based on the suggested values in the public software package (penalty parameters *β*_1_ = 10^−9^ for Schatten and *β*_2_ = 10^−2^ for TV norms, maximum number of 50 inner and 9 outer iterations). In *k-t* RPCA, two regularization parameters are *μ* = 200 and *ρ* = 1.5 for the regularization and decomposition, respectively.

Similarly, the method requires the specification of three parameters *λ*, *ρ*, and *μ*. We set *μ*_0_ = 1.5/‖**X**‖_2_ and *ρ* = 1.2. We may take ‖**X** − **A**_*k*_ − **E**_*k*_‖_*F*_/‖**X**‖_*F*_ < 10^−7^ as the stopping criteria for Algorithm 1. We chose a fixed weight parameter λ = max(*n*_*x*_∗*n*_*y*_, *n*_*t*_)^−1/2^ by the suggestion of the authors of [16]. The proposed algorithm was verified by experiments using the fully sampled cardiac cines (two mentioned datasets above) with two different sampling trajectories.

**Figure 2:**
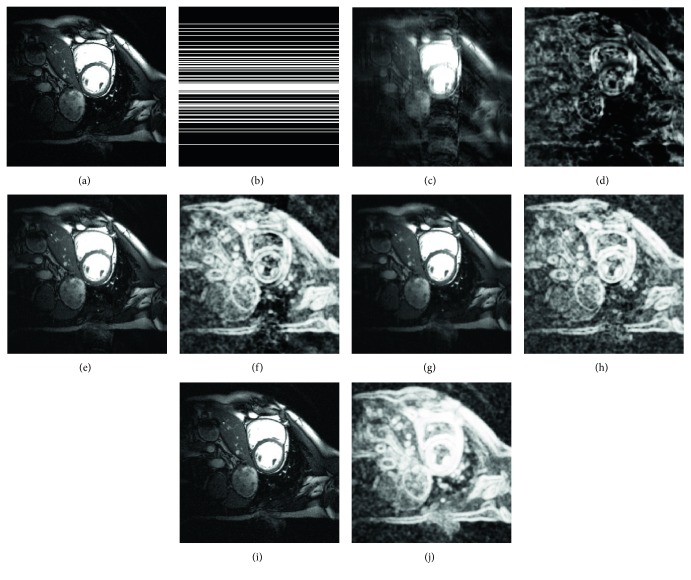
Comparison of the reconstruction results with different methods on the first cardiac dataset. The acceleration rate is 4 or sampling ratio is about 0.25. Fully sampled image (a) and undersampling mask (b), undersampled by zero-filled directly (c), reconstructions using *k-t* RPCA (e), *k-t* SLR (g), and proposed method (i) with their respective residuals (d, f, h, j).

**Figure 3:**
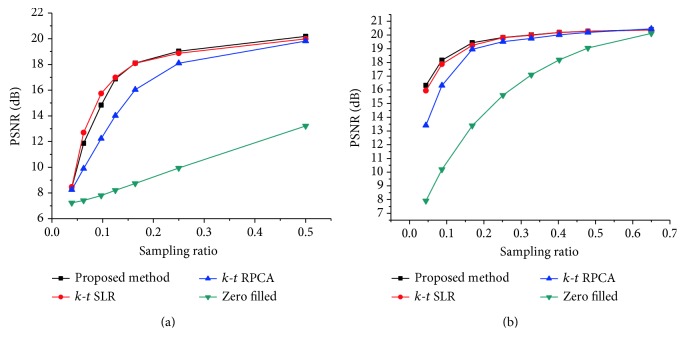
PSNR performance of different reconstructions evaluated versus the sample ratio for the first cardiac MRI dataset with Cartesian sampling (a) and radial sampling (b).

**Figure 4:**
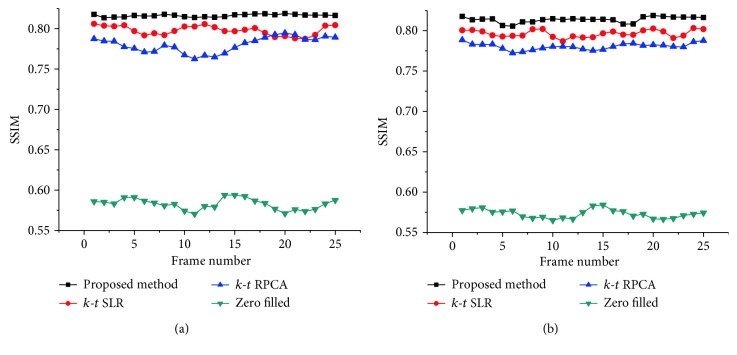
SSIM performance of different reconstructions versus each time frame for the first cardiac MRI dataset at acceleration factor 6 with Cartesian sampling (a) and radial sampling (b).

For simulating the acceleration of the *k*-space, the fully sampled *k*-space data was artificially subsampled by using variable density (sampling factor) random sampling. To test the robustness of the proposed method, the *k*-space data of the two datasets are corrupted with additional complex Gaussian white noise with fixed standard deviation *σ* = 15. Firstly, this method was tested on the first cardiac dataset by using different sampling models with variable sampling ratio. A comparison of the visual quality was showed in Figure 2, which compares the reconstruction results between the proposed method (Algorithm 1) and the other methods. The acceleration factor is approximately 4 (about 25% of acquired samples) for Cartesian sampling masks.  Figure 3 shows the PSNR of the reconstructed results for Cartesian sampling and pseudo-radial sampling as a function of sampling factor. It can be seen that the performance of the proposed method outperforms the other two methods with pseudo-radial sampling acquisition. But the performance at lower sampling ratio is slightly lower than the *k-t* SLR method with Cartesian sampling. Additionally, SSIM at each time frame is shown in Figure 4 for both Cartesian and radial sampling at the same sampling factor (an acceleration factor of approximately 6 with about 16.4% of acquired samples). The experimental results reveal that the proposed method achieved a superior reconstruction result in terms of SSIM and hence the advantage of our method is more relatively evident when pseudo-radial sampling is used instead of Cartesian sampling.

**Figure 5:**
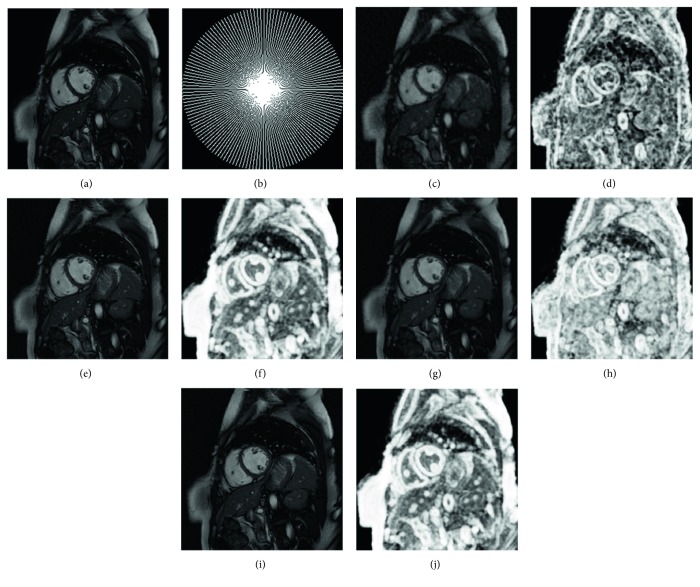
Comparison of the reconstruction results with different methods on the second cardiac dataset. The number of radial trajectory is 74 and sampling ratio is about 0.25. Fully sampled image (a) and undersampling mask (b), undersampled by zero-filled directly (c), reconstructions using *k-t* RPCA (e), *k-t* SLR (g), and proposed method (i) with their respective residuals (d, f, h, j).

**Figure 6:**
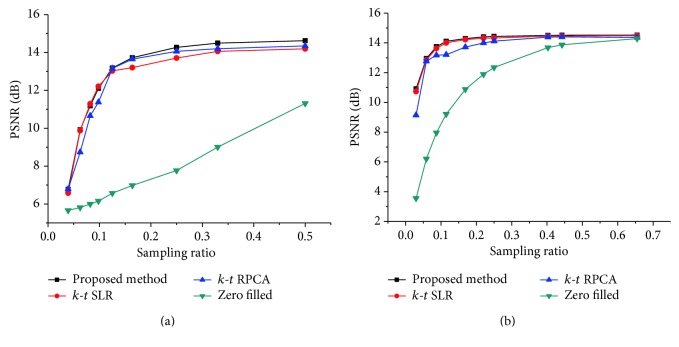
PSNR performance of different reconstructions evaluated versus the sample ratio for the second cardiac MRI dataset with Cartesian sampling (a) and radial sampling (b).

Moreover, we tested our proposed method on the second cardiac dataset by using same experimental method.  Figure 5 provides the visual evaluations for radial sampling with an acceleration rate of 4 (about 25% of acquired samples). Quantitative results (PSNR performance) are reported for Cartesian sampling and pseudo-radial sampling in Figure 6. It is observed that the performance of reconstructions by using the two sampling models is similar to Figure 3.

Figures 3 and 6 indicate that both the proposed and the other two methods are effective to the choice of Cartesian sampling with higher sampling ratios. However, the choice of pseudo-radial sampling ensures that the greater performance can be obtained at lower sampling ratios. Moreover, it can be acquired that the proposed method is more robust in a cardiac cycle.

We also evaluated the execution time of the three methods by using different sampling models with variable sampling factors on different datasets.  Table 1 shows the average computational time for reconstructing the cardiac MRI images in complete temporal frames. From the Table 1, it can be known that our method is faster than the other two methods, and it is more potential for online dMRI reconstruction.

## 5. Conclusion

In this paper, we proposed a scalable and fast algorithm (IALM) for solving RPCA optimization problem to recover dMRI sequence from highly undersampled *k*-space data. Our proposed algorithm has a generalized formulation capability of separating dynamic MR data into low-rank component and sparse component. And this algorithm reconstructs and separates simultaneously dynamic MR data from partial measurement. Experiments on cardiac datasets have validated the efficiency and effectiveness compared to the state-of-the-art methods.

## Figures and Tables

**Algorithm 1:**
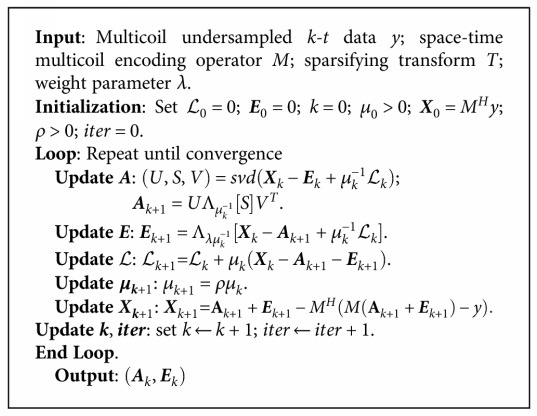
Algorithm 1: The proposed algorithm.

**Table 1:** Average computational time for cardiac datasets in complete temporal frames (seconds).

Method	Cardiac dataset 1 (256 × 256 × 25)		Cardiac dataset 2 (256 × 256 × 30)	
	Cartesian sampling	Pseudo-radial sampling	Cartesian sampling	Pseudo-radial sampling
*k-t* SLR	1676.7	1391.5	1456.3	1515.7
*k-t* RPCA	610.3	766.6	423.3	681.2
Proposed method	131.2	124.6	116.2	130.6

## References

[1] Lai Z., Qu X., Liu Y. (2016). Image reconstruction of compressed sensing MRI using graph-based redundant wavelet transform. *Medical Image Analysis*.

[2] Sodickson D. K., Manning W. J. (1997). Simultaneous acquisition of spatial harmonics (SMASH): fast imaging with radiofrequency coil arrays. *Magnetic Resonance in Medicine*.

[3] Larkman D. J., Nunes R. G. (2007). Parallel magnetic resonance imaging. *Physics in Medicine and Biology*.

[4] Pruessmann K. P., Weiger M., Scheidegger M. B., Boesiger P. (1999). SENSE: sensitivity encoding for fast MRI. *Magnetic Resonance in Medicine*.

[5] Lustig M., Donoho D., Pauly J. M. (2007). Sparse MRI: the application of compressed sensing for rapid MR imaging. *Magnetic Resonance in Medicine*.

[6] Ravishankar S., Bresler Y. (2011). MR image reconstruction from highly undersampled k-space data by dictionary learning. *IEEE Transactions on Medical Imaging*.

[7] Huang Y., Paisley J., Lin Q., Ding X., Fu X., Zhang X. P. (2014). Bayesian nonparametric dictionary learning for compressed sensing MRI. *IEEE Transactions on Imaging Processing*.

[8] Donoho D. L. (2006). Compressed sensing. *IEEE Transactions on Information Theory*.

[9] Jung H., Sung K., Nayak K. S., Kim E. Y., Ye J. C. (2009). k-t FOCUSS: a general compressed sensing framework for high resolution dynamic MRI. *Magnetic Resonance in Medicine*.

[10] Lingala S. G., Hu Y., DiBella E., Jacob M. (2011). Accelerated dynamic MRI exploiting sparsity and low-rank structure: k-t SLR. *IEEE Transactions on Medical Imaging*.

[11] Awate S. P., DiBella E. V. R. Spatiotemporal dictionary learning for undersampled dynamic MRI reconstruction via joint frame-based and dictionary-based sparsity.

[12] Caballero J., Price A. N., Rueckert D., Hajnal J. V. (2014). Dictionary learning and time sparsity for dynamic MR data reconstruction. *IEEE Transactions on Medical Imaging*.

[13] Majumdar A., Ward R. K. Learning the sparsity basis in low-rank plus sparse model for dynamic MRI reconstruction.

[14] Majumdar A., Ward R. K. (2012). Exploiting rank deficiency and transform domain sparsity for MR image reconstruction. *Magnetic Resonance Imaging*.

[15] Otazo R., Candès E., Sodickson D. K. (2015). Low-rank and sparse matrix decomposition for accelerated dynamic MRI with separation of background and dynamic components. *Magnetic Resonance in Medicine*.

[16] Trémoulhéac B., Dikaios N., Atkinson D., Arridge S. R. (2014). Dynamic MR image reconstruction-separation from undersampled (k,t)-space via low-rank plus sparse prior. *IEEE Transactions on Medical Imaging*.

[17] Majumdar A., Ward R. K., Aboulnasr T. (2013). Non-convex algorithm for sparse and low-rank recovery: application to dynamic MRI reconstruction. *Magnetic Resonance Imaging*.

[18] Majumdar A. (2013). Improved dynamic MRI reconstruction by exploiting sparsity and rank-deficiency. *Magnetic Resonance Imaging*.

[19] Gamper U., Boesiger P., Kozerke S. (2008). Compressed sensing in dynamic MRI. *Magnetic Resonance in Medicine*.

[20] Haldar J. P., Liang Z. P. Spatiotemporal imaging with partially separable functions: a matrix recovery approach.

[21] Hu Y., Lingala S. G., Jacob M. (2012). A fast majorize-minimize algorithm for the recovery of sparse and low-rank matrices. *IEEE Transactions on Imaging Processing*.

[22] Zhao B., Haldar J. P., Christodoulou A. G., Liang Z. P. (2012). Image reconstruction from highly undersampled-space data with joint partial separability and sparsity constraints. *IEEE Transactions on Medical Imaging*.

[23] Lingala S. G., Jacob M. (2013). Blind compressive sensing dynamic MRI. *IEEE Transactions on Medical Imaging*.

[24] Lebel R. M., Jones J., Ferre J. C., Law M., Nayak K. S. (2014). Highly accelerated dynamic contrast enhanced imaging. *Magnetic Resonance in Medicine*.

[25] Candès E., Recht B. (2009). Exact matrix completion via convex optimization. *Foundations of Computation Mathematics*.

[26] Zhou Z., Li X., Wright J., Candes E., Ma Y. Stable principal component pursuit.

[27] Lin Z., Chen M., Ma Y. The augmented Lagrange multiplier method for exact recovery of corrupted low-rank matrices. http://arxiv.org/abs/1009.5055.

[28] Cai J. F., Candès E. J., Shen Z. (2010). A singular value thresholding algorithm for matrix completion. *SIAM Journal on Optimization*.

[29] Wang Z., Bovik A. C., Sheikh H. R., Simoncelli E. P. (2004). Image quality assessment: from error visibility to structural similarity. *IEEE Transaction on Image Processing*.

